# Obtaining structural insights into metal-organic frameworks and covalent organic frameworks using three-dimensional electron diffraction

**DOI:** 10.1107/S2052252526009541

**Published:** 2026-11-09

**Authors:** Lijuan Wang, Zhehao Huang

**Affiliations:** a School of Emergent Soft Matter, Center for Electron Microscopy, State Key Laboratory of Luminescent Materials and Devices South China University of Technology Guangzhou Guangdong510640 People’s Republic of China; b Guangdong Basic Research Center of Excellence for Energy and Information Polymer Materials, Guangdong Provincial Key Laboratory of Functional and Intelligent Hybrid Materials and Devices, Guangzhou510640, People’s Republic of China; Istituto Italiano di Tecnologia, Italy

**Keywords:** three-dimensional electron diffraction, 3D ED, metal–organic frameworks, MOFs, covalent organic frameworks, COFs, structural analysis

## Abstract

Metal–organic frameworks (MOFs) and covalent organic frameworks (COFs) have attracted great interest owing to their high surface areas and tunable pore structures. Understanding their structures at the atomic level is essential for revealing structure–property relationships and exploring their applications. However, many MOFs and COFs are obtained as nanocrystals or polycrystalline powders, making structure determination by conventional X-ray diffraction difficult. Three-dimensional electron diffraction (3D ED) has emerged as a powerful approach for single-crystal structure analysis from nanocrystals. In this review, we summarize recent progress in the application of 3D ED to MOFs and COFs, including discovery and structural determination of new materials, and revealing structural insights such as framework flexibilities, host–guest interactions and dynamic structural behaviors.

## Abstract 2

Three-dimensional electron diffraction (3D ED) has been developed for structural analysis of metal–organic frameworks (MOFs) and covalent organic frameworks (COFs) that are inaccessible to conventional X-ray methods. This review highlights recent advances in applying 3D ED to framework materials, including discovery and structure determination of novel MOFs and COFs, and obtaining chemical insights.

## 1. Introduction

Metal–organic frameworks (MOFs) (Li *et al.*, 1999 ▸; Kondo *et al.*, 1997 ▸) and covalent organic frameworks (COFs) (Côté *et al.*, 2005 ▸; El-Kaderi *et al.*, 2007 ▸) are two important classes of crystalline materials that can be rationally designed through the assembly of molecular building units into extended porous structures. Compared with conventional porous materials (Lee *et al.*, 2006 ▸; Zhou *et al.*, 2025 ▸; Xue & Yan, 2025 ▸), MOFs and COFs have high surface areas, as well as tunable pore structures and connectivities, making them attractive for applications in catalysis (Pascanu *et al.*, 2019 ▸; Guo & Jiang, 2020 ▸; Stanley *et al.*, 2022 ▸), gas capture and separation (Chanut *et al.*, 2020 ▸; Wang *et al.*, 2022 ▸), biomedicine (McKinlay *et al.*, 2010 ▸; Tyagi *et al.*, 2023 ▸; Abánades Lázaro *et al.*, 2024 ▸), sensing (Gilmanova *et al.*, 2021 ▸; Krämer *et al.*, 2022 ▸) and other fields (Li *et al.*, 2020 ▸; Moghadam *et al.*, 2024 ▸). Determination of their crystal structures is key to understanding their properties, establishing structure–property relationships, and guiding the design of new framework materials. However, many MOFs and COFs can only be obtained as nanocrystalline materials. In addition to their limited crystal dimensions, MOFs and COFs are highly sensitive to electron beam irradiation (Forgan, 2020 ▸; Liu *et al.*, 2024 ▸), making atomic-level structure determination by transmission electron microscopy (TEM) challenging.

At present, single-crystal X-ray diffraction (SCXRD), powder X-ray diffraction (PXRD) and electron diffraction are the main methods used for crystal structure determination of MOFs and COFs. Among them, SCXRD requires sufficiently large and well ordered crystals, which are often demanding to obtain for many framework materials (Metherall *et al.*, 2023 ▸). PXRD is widely used for polycrystalline samples, but severe peak overlap, especially for MOFs and COFs with large unit cells, low symmetry and multiple phases, makes structure determination challenging. Such limitations have led to a bottleneck in the characterization and development of MOFs and COFs.

Compared with X-rays, electrons interact much more strongly with matter, allowing diffraction data with sufficient signal-to-noise ratios to be collected from nano-sized crystals. Since its first development, different variations of 3D ED protocols have been established. Early 3D ED methods such as automated diffraction tomography (ADT) (Kolb *et al.*, 2007 ▸), rotation electron diffraction (RED) (Zhang *et al.*, 2010 ▸) and electron diffraction tomography (EDT) (Gemmi & Oleynikov, 2013 ▸) have been developed with the focus on analyzing structures from nanocrystals. By collecting data in 3D reciprocal space while tilting crystals away from main zone axes, 3D ED can reduce dynamical effects (Gemmi *et al.*, 2019 ▸). Further developments have adopted a continuously tilting TEM goniometer instead of step-wise tilting (Nannenga *et al.*, 2014 ▸; Gemmi *et al.*, 2015 ▸; Gruene *et al.*, 2018 ▸; Jones *et al.*, 2018 ▸; Cichocka *et al.*, 2018 ▸; Gemmi *et al.*, 2019 ▸; Cichocka *et al.*, 2020 ▸), and precession of the electron beam has been utilized to reduce dynamical effects (Boullay *et al.*, 2013 ▸; Shen *et al.*, 2022 ▸). These recent 3D ED developments not only improved the accuracy of diffraction intensities but also significantly reduced the required electron dose for collecting a whole dataset. Furthermore, the introduction of the dynamical refinement method tackles dynamical effects in electron diffraction data and has improved the accuracy of 3D ED (Palatinus *et al.*, 2015 ▸; Klar *et al.*, 2023 ▸).

**Figure 1:**
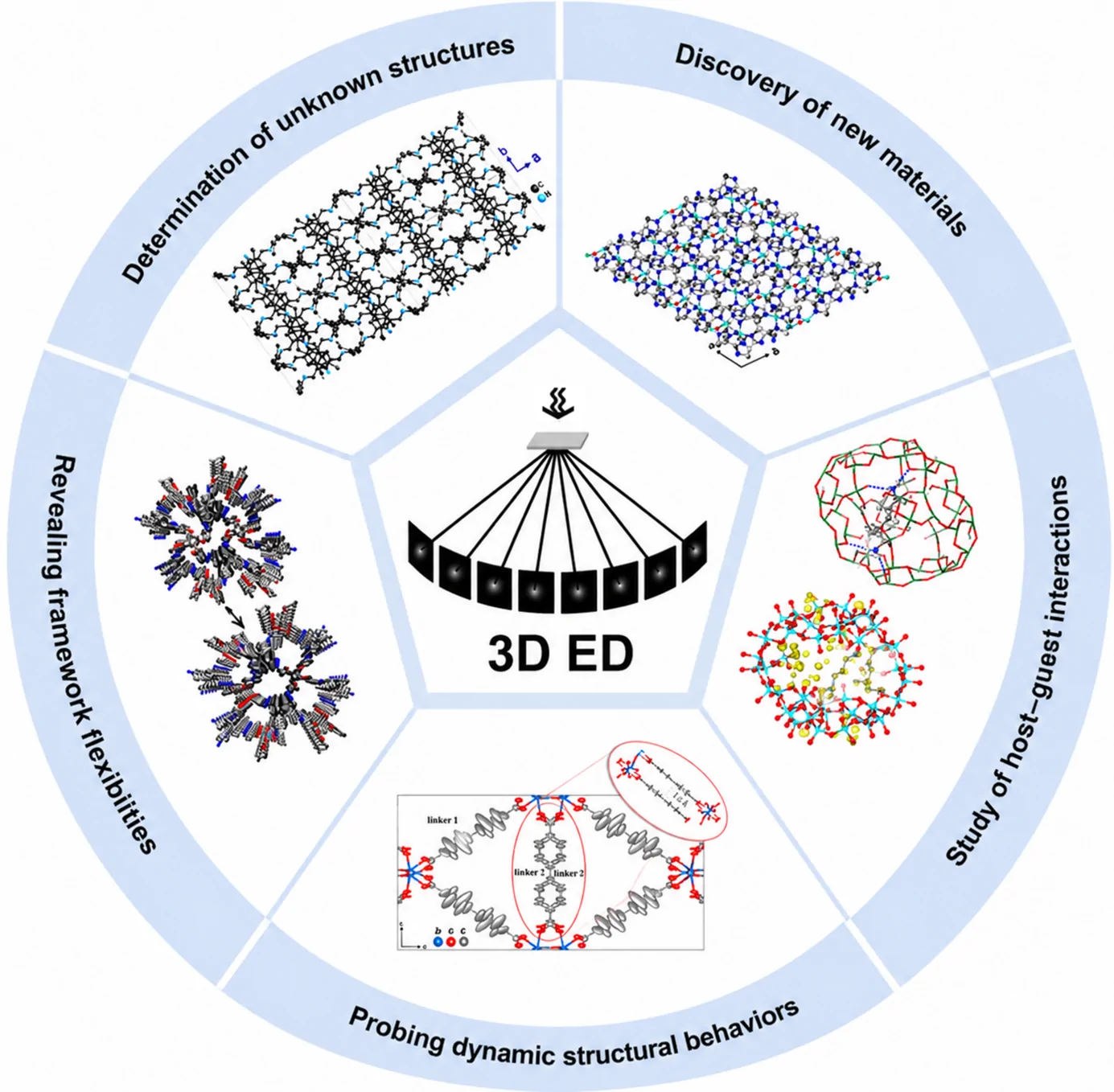
Schematic summary of applying 3D ED for the studies of MOFs and COFs.

In this review, we summarize the advances in applying 3D ED for structural analysis of MOFs and COFs. We focus on using 3D ED for determination of unknown structures, discovery of new materials, study of host–guest interactions, revealing framework flexibilities and probing dynamic structural behaviors (Fig. 1 ▸). By analyzing structural details at the atomic level, 3D ED allows chemical insights to be obtained that could be challenging for other characterization methods. In addition, the limitations and future prospects of applying 3D ED to MOFs and COFs are discussed.

## 2. Applications in MOFs and COFs

### 2.1. Determination of unknown structures

**Figure 2:**
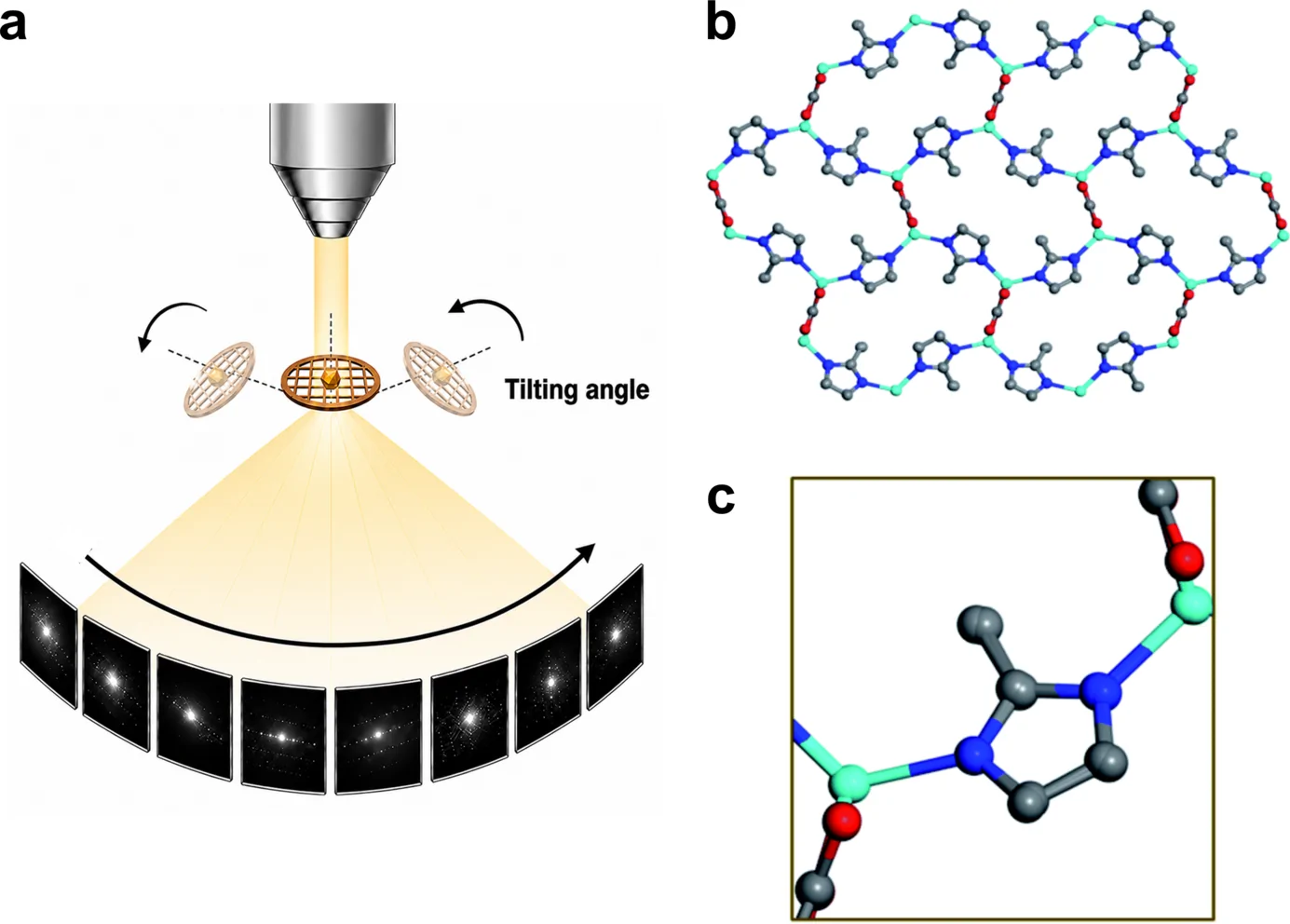
(*a*) Schematic illustration of continuous-tilting-based 3D ED methods. Comparison of the overlayed structures of ZIF-CO_3_-1 obtained by 3D ED and SCXRD, (*b*) viewed along the *c* axis and (*c*) enlarged view of the 2-methyl­imidazolate linker and Zn(II) sites. Parts (*b*) and (*c*) reprinted from Huang, Ge *et al.* (2021 ▸).

Recent methodological advances have established 3D ED as a robust crystallographic method for structure determination from nanocrystals (Gruene *et al.*, 2021 ▸; Gemmi *et al.*, 2015 ▸), and the continuous-tilting-based methods such as microcrystal electron diffraction (MicroED), fast electron diffraction tomography (Fast-EDT) *etc*. have been proven as powerful tools, especially for structure determination of unknown MOFs and COFs (Nannenga & Gonen, 2019 ▸; Huang, Grape *et al.*, 2021 ▸; Yang *et al.*, 2022 ▸; Sala *et al.*, 2024 ▸). In such methods, the crystal is continuously tilted under electron-beam illumination while diffraction patterns are continuously recorded, enabling integration of reflection intensities for structure determination [Fig. 2 ▸(*a*)]. Importantly, the continuous tilting significantly reduces the data collection time to 1–5 minutes. As the electron dose applied to a crystal is proportional to the electron beam illumination time, the reduced data collection time effectively reduces the electron dose applied to the crystal. This is crucial for MOFs and COFs, which contain large amounts of organic species, making them highly sensitive to electron irradiation and susceptible to structural collapse or amorphization under high electron doses (Yang *et al.*, 2022 ▸; Bardin *et al.*, 2024 ▸; Xu *et al.*, 2025 ▸). In addition, collecting 3D ED data at cryogenic temperatures could reduce heating effects, suppress the diffusion of beam-generated radicals, and mitigate electron beam damage applied to MOFs and COFs (Banihashemi *et al.*, 2020 ▸; Samperisi *et al.*, 2021 ▸; Li *et al.*, 2022 ▸).

As an emerging method in the recent decade, the accuracy of applying 3D ED to study MOFs and COFs has drawn great interest, especially for those complex structures that are difficult for dynamical refinement and using the kinematical assumption. The reliability of 3D ED has been validated by using MOFs PCN-415 and PCN-416, which are based on [Ti_8_Zr_2_O_12_(COO)_16_] clusters, and 1,4-benzene­dicarboxyl­ate (BDC) and 2,6-naphthalenedi­carboxyl­ate (NDC) linkers, respectively (Yuan, Qin, Xu *et al.*, 2018 ▸). Comparing the structural models refined against 3D ED by the least-squares method and against PXRD by the Rietveld method showed excellent agreement in atomic positions, with average deviations of 0.032 Å for the Zr/Ti atoms and 0.071 Å for the O/C atoms, confirming the high reliability of 3D ED. Further investigation of the structural accuracy obtained from 3D ED compared with PXRD has been made on the isostructural Co(C_8_H_4_O_4_)·2H_2_O (CSD refcode BUKLOJ03) and Mn(C_8_H_4_O_4_)·2H_2_O (XUDSUK01) frameworks (Biehler *et al.*, 2025 ▸). The differences in Co—O and Mn—O bond lengths within the metal coordination octahedra determined by the two approaches were below 2%. In addition, direct comparison between 3D ED and synchrotron SCXRD results for ZIF-CO_3_-1 (XADNUP) confirmed the accuracy of models obtained by 3D ED (Huang, Ge *et al.*, 2021 ▸). It revealed that it was difficult to distinguish two models in which the Zn positions differed by 0.06(1) Å, while the average deviations for light-atom positions and bond lengths were 0.07(3) and 0.04(3) Å, respectively [Figs. 2 ▸(*b*) and 2 ▸(*c*)]. These studies suggest that 3D ED can provide structural models with an accuracy comparable to X-ray diffraction, despite the high residual factors often associated with dynamical effects.

**Figure 3:**
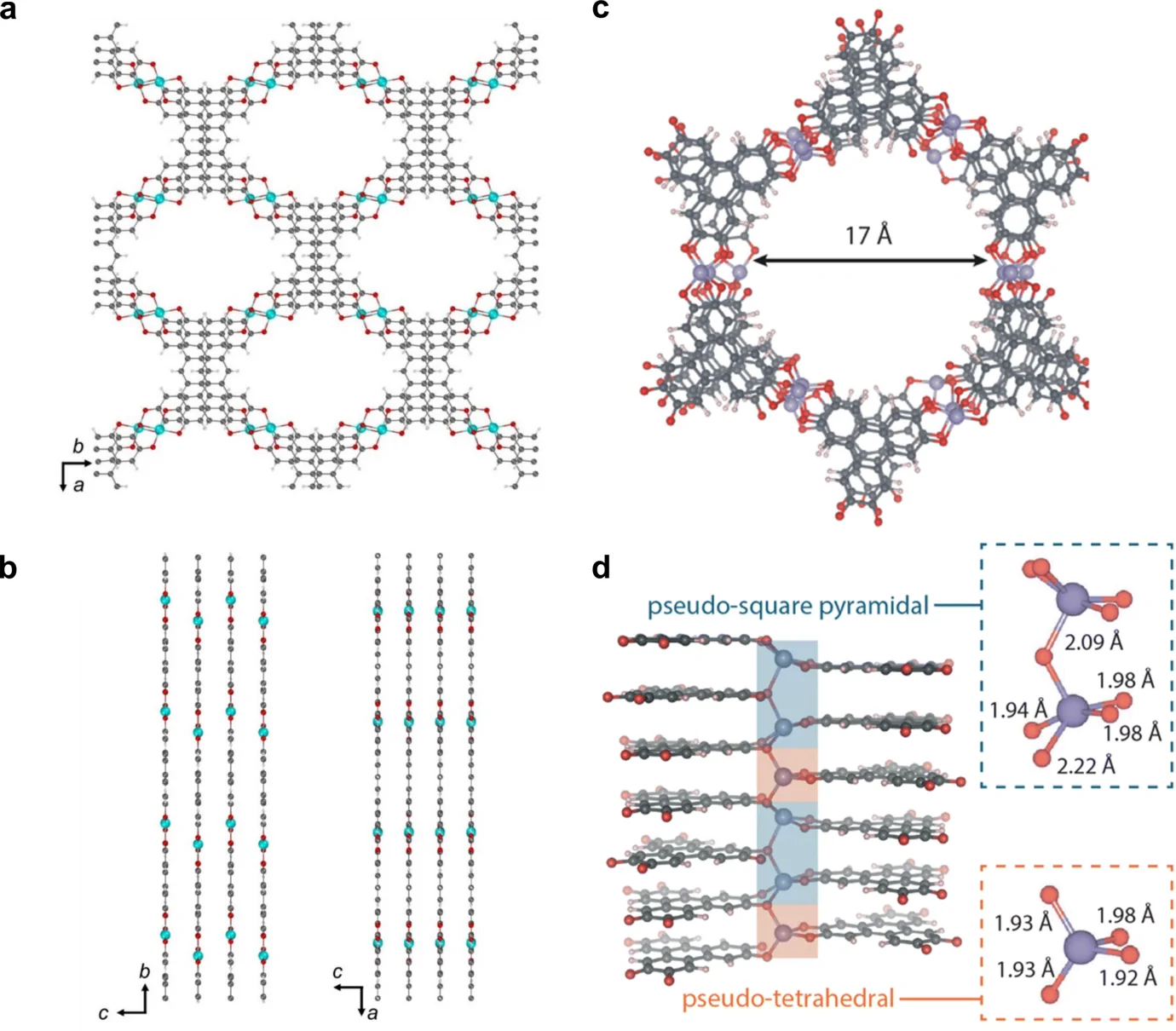
Structural model of Cu_2_(OHPTP) viewed along (*a*) the *c* axis and (*b*) the *a* axis and the *b* axis. (*c*) Structural model of Zn_3_(HOTP)_2_ viewed along the *c* axis. (*d*) Commensurate approximant model showing pseudo-square-pyramidal (blue) and pseudo-tetrahedral (orange) Zn(II) coordination environments. Parts (*a*) and (*b*) reprinted from Sporrer *et al.* (2023 ▸). Copyright (2023) Wiley-VCH GmbH. Parts (*c*) and (*d*) reprinted from Zhang, Chen *et al.* (2025).

3D ED has increasingly been employed for *ab initio* structure determination of unknown MOFs that are inaccessible to conventional approaches (Feyand *et al.*, 2012 ▸; Leubner *et al.*, 2019 ▸; Banihashemi *et al.*, 2020 ▸; Ling *et al.*, 2022 ▸; Zhang, Chen *et al.*, 2025 ▸). It is particularly valuable for 2D MOFs, because it can resolve both in-plane structure and interlayer stacking, which is difficult to distinguish by PXRD owing to peak broadening and overlap (Dou *et al.*, 2021 ▸; Chen *et al.*, 2022 ▸; Sporrer *et al.*, 2023 ▸). For instance, 3D ED was used to determine the structure of a 2D MOF, Cu_3_(HHTT)_2_ (HHTT = 2,3,7,8,12,13-hexa­hydroxy­tetra­aza­naphtho­tetraphene), which was constructed from Cu(II) ions and the π-conjugated ligand HHTT (Dou *et al.*, 2021 ▸). 3D ED revealed that the square-planar [CuO_4_] secondary building units are linked by HHTT ligands to form an extended honeycomb-like 2D network. In addition, it showed an eclipsed AA stacking of adjacent layers, with a short interlayer distance of 3.19 Å. Such close and ordered stacking promotes strong interlayer electronic coupling and efficient orbital overlap between neighboring HHTT layers. As a result, charge transport is facilitated not only within the in-plane π-conjugated framework but also along the out-of-plane direction, leading to highly dispersive electronic bands and enhanced electrical conductivity. Another 2D MOF, Cu_2_(OHPTP) (XIKHOS) (OHPTP = 2,3,6,7,11,12,15,16-octa­hydroxy­phenanthro[9,10-*b*]tri­phenyl­ene), featured square-planar CuO_4_ coordination units and the *D*_2*h*_-symmetry ligand OHPTP (Sporrer *et al.*, 2023 ▸) [Fig. 3 ▸(*a*)]. A distinctive structural arrangement revealed by 3D ED showed an unusual slipped AA stacking of adjacent layers [Fig. 3 ▸(*b*)]. Unlike conventional eclipsed stacking, neighboring layers are shifted by approximately 2 Å along the *b* axis while maintaining a short interlayer distance of ∼3.2 Å, resulting in displaced π–π overlap between aromatic cores. Such stacking behavior strengthens interlayer electronic coupling and promotes charge delocalization along the stacking direction. Consequently, the strong interlayer orbital interactions contribute significantly to the semiconducting behavior and high carrier mobility of Cu_2_(OHPTP), highlighting the critical role of understanding layer stacking behavior by 3D ED.

While 3D ED has been employed to reveal detailed stacking modes of 2D MOFs, it has also proven instrumental in revising the structures of MOFs with higher structural complexity. 3D ED revealed that Zn_3_(HOTP)_2_ (RAFFOY) (HOTP = hexaoxidotri­phenyl­ene), a 3D conductive MOF composed of Zn(II) nodes and HOTP linkers, adopted a 3D framework rather than the previously assumed layered 2D structure that resulted from PXRD analysis [Figs. 3 ▸(*c*) and 3 ▸(*d*)]. This structural revision was crucial for interpreting its charge-transport behavior and incommensurate modulation, thereby highlighting the capability of 3D ED to resolve complex and previously overlooked framework structures (Zhang, Chen *et al.*, 2025 ▸). More recently, the conductive 2D MOF Cu-HHTP (WAGBOA) (HHTP = 2,3,6,7,10,11-hexa­hydroxy­tri­phenyl­ene), whose crystal structure had remained uncertain because of its micro- to nanocrystalline nature, was elucidated by 3D ED (Marchetti *et al.*, 2026 ▸). The structure revealed a two-dimensional honeycomb framework with slipped π–π stacked layers and one-dimensional channels, providing an unambiguous structural model. Furthermore, the structural model guided the optimization of a mechanochemical synthesis, illustrating how accurate structural information obtained by 3D ED can support the rational development of functional conductive MOFs.

**Figure 4:**
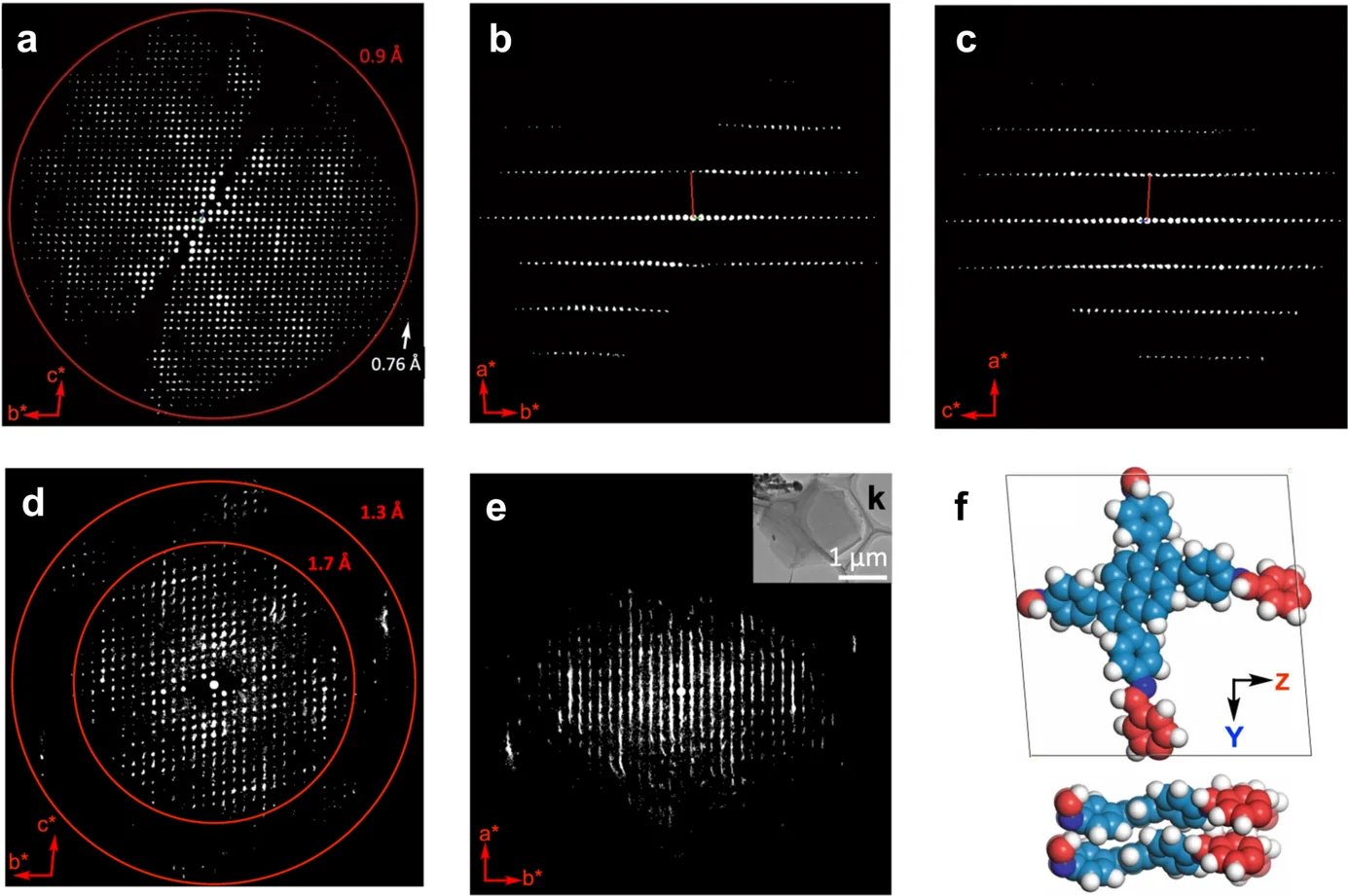
3D reciprocal lattice of Py-IP viewed along (*a*) the [100] direction, (*b*) the [001] direction and (*c*) the [010] direction. 3D reciprocal lattice of the Py-IP-6 structure viewed along (*d*) the [100] direction and (*e*) the [001] direction. (*f*) The structural model of Py-IP. Reprinted from Kang *et al.* (2022 ▸).

Owing to the low dose requirement, 3D ED has provided a crucial route for *ab initio* structure determination of covalent organic frameworks (COFs), which are often challenging because of their limited crystallinity and increased beam sensitivity (Liu *et al.*, 2021 ▸; Xiao *et al.*, 2023 ▸; Yu *et al.*, 2024 ▸; Zeng *et al.*, 2024 ▸; Chang *et al.*, 2025 ▸). For example, 3D ED was applied to determine the single-crystal structure of Py-IP (PAXYEW) (Kang *et al.*, 2022 ▸). It showed that Py-IP was an imine-linked 2D COF constructed from 4,4′,4″,4‴-(1,9-di­hydro­pyrene-1,3,6,8-tetrayl)-tetraaniline (DTA) and (1,4-phenyl­ene)bis­(*N*-phenyl­methanimine) (PPA). The 2D layers were stacked in an AA-fashion through π–π interactions [Figs. 4 ▸(*a*–*c*) and 4(*f*)]. Meanwhile, 3D ED revealed that the majority of the structures exhibited a six-layer stacking arrangement, in which six individual layers were periodically stacked within one unit cell [Figs. 4 ▸(*d*) and 4 ▸(*e*)]. This finding demonstrated that the growth and stacking behavior of COFs can be significantly more complex than had been widely assumed, and that multiple stacking modes may coexist within a single COF growth system.

More recently, 3D ED was employed to determine the atomically resolved structures of three single-crystal 2D COF isomers, GZU-1 (EXULUI), GZU-2 (EYEJIF01) and GZU-3 (EYEJIF), constructed through imine condensation of sub­stituted 1,3,5-tris­(phenyl)­benzene-based amine and aldehyde building blocks (Yu *et al.*, 2026 ▸). The structures revealed hcb-b honeycomb layers with distinct imine orientations and previously unobserved stacking modes, including an ABCDEF sixfold alternating stacking (AAl_6_) structure and a unidirectional ABCD fourfold tilted stacking (AAt_4_) structure. These structural insights established direct correlations between interlayer packing, permanent porosity and methane storage performance, further demonstrating the capability of 3D ED in determining previously unknown 2D COF structures and their structure–property relationships.

Furthermore, 3D ED was applied to the structural elucidation of the 3D COF JUC-644 (KIYRET), which was con­structed from the reduced-symmetry 8-connected building blocks 3,3′,5,5′-tetra(3″,5″-di­formyl­phenyl)-bimesityl­ene (TDFBM) together with the linear linker *p*-phenyl­enedi­amine (PDA) (Chang *et al.*, 2025 ▸). The 3D ED data allowed the *ab initio* structure determination of JUC-644 at a resolution of 1.5 Å, revealing the microporous architecture of JUC-644 and the presence of well defined pore channels within the twofold interpenetrated framework. It was further discovered that the 8-connected building blocks in these structures exhibited an unexpected linkage of six, because some of the linkers are connected through two positions. This structural information provided a basis for understanding JUC-644’s high adsorption capacities for C_3_H_8_ and *n*-C_4_H_10_, as well as its excellent C_3_H_8_/C_2_H_6_ and *n*-C_4_H_10_/C_2_H_6_ separation performance.

A recent study has further extended the application of 3D ED to metal–organic and covalent organic framework (MOCOF) hybrid materials (Zhang, Zhong *et al.*, 2025 ▸). Structural analysis of MOCOF-113 (YURPEK) by 3D ED revealed that the [5,10,15,20-tetra­kis­(4-amino­phenyl)­porphinato]iron [Fe(TAP)] porphyrin units were linked to 1,4-benzene­dicarboxaldehyde (BDA) through imine condensation and formed 1D covalent chains along the *b* axis. Simultaneously, the adjacent Fe centers were connected by 5,10,15,20-tetra(4-pyridyl)­porphyrin (TPP) ligands to generate a second set of 1D coordination chains along the *c* axis. The cross-linking of these covalent and coordination chains gave rise to a 2D square network, integrating COF- and MOF-type connectivities within a single crystalline architecture. 3D ED revealed that the slip-stacked architecture contained closely spaced porphyrin macrocycles. They were subsequently metalated with Pt(II) to generate twin Pt-porphyrin catalytic sites. The well defined arrangement of these neighboring Pt-porphyrin centers facilitates cooperative CO_2_ activation and stabilizes reaction intermediates within the framework channels, thereby promoting C—C coupling and guiding the reaction pathway toward ethanol production, which contributes to the enhanced photoelectrochemical CO_2_ reduction performance.

**Figure 5:**
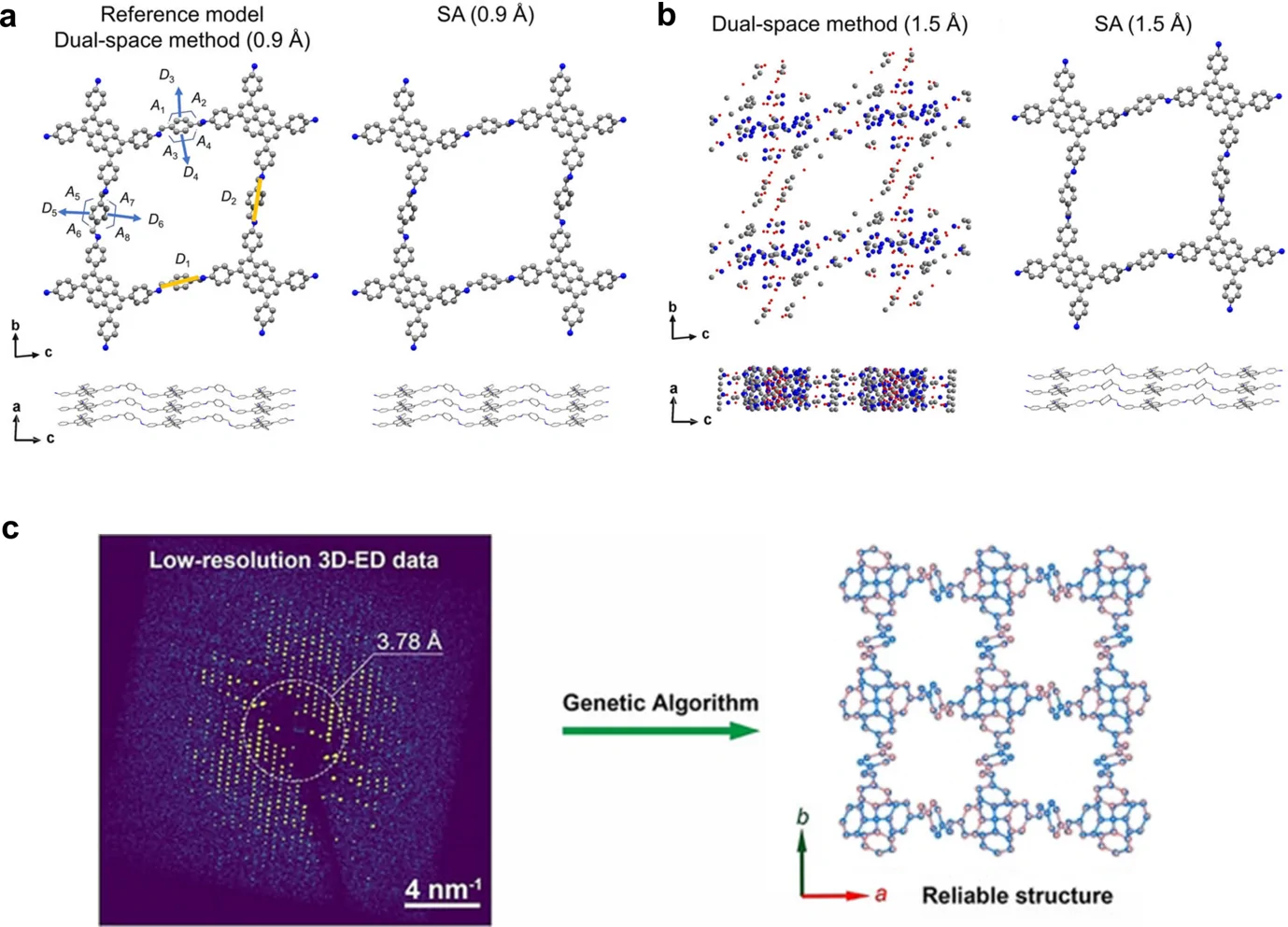
Structural models of Py-IP obtained using the dual-space and SA methods at (*a*) 0.9 Å resolution and (*b*) 1.5 Å resolution. (*c*) Structure determination of COF-300 from low-resolution 3D ED data using GA. Parts (*a*) and (*b*) reprinted from Zhou *et al.* (2023 ▸). Part (*c*) reprinted with permission from Sun *et al.* (2020 ▸). Copyright (2020) Wiley-VCH GmbH.

Although 3D ED has been successfully applied to the direct determination of unknown MOF and COF structures, it typically requires a data resolution of 1.2 Å. However, in those cases where synthesis of MOFs and COFs cannot provide such high-quality crystals, low resolution 3D ED data can still provide important structural information such as unit-cell parameters, space groups and stacking characteristics along specific directions. Moreover, recent advances show that real-space-based structure solution methods using 3D ED data can provide more structural details. For example, simulated annealing (SA) against 3D ED data has been employed for solving the COF Py-IP structure with low data resolution (Zhou *et al.*, 2023 ▸). Compared with the resulting model obtained from 0.9 Å data resolution, it was found that the COF Py-IP structural model obtained by SA was in good agreement with the refined model obtained by a dual-space method [Figs. 5 ▸(*a*) and 5 ▸(*b*)]. When the crystallinity decreased and the resolution of 3D ED data was 1.5 Å, the dual-space method could no longer provide an accurate structural model, yet structure solution by SA was still successful. For MOFs, the structural model can also be established by combining 3D ED data with SA methods (Zhang *et al.*, 2023 ▸). This strategy was successfully applied to the MOF Cu_3_(HFcHBC)_2_ (HFcHBC = 2,3,10,11,18,19-hexa­fluoro-6,7,14,15,22,23-hexa­hydroxy contorted hexa-cata-hexabenzocoronene). In this work, 3D ED data with a resolution of 1.8 Å were used to successfully obtain the structural model through simulated annealing. The resolved structure revealed a wavy honeycomb structure with AA-eclipsed stacking. The combination of 3D ED and simulated annealing can provide reliable structural insights into MOFs with unusual structures and limited diffraction information.

**Figure 6:**
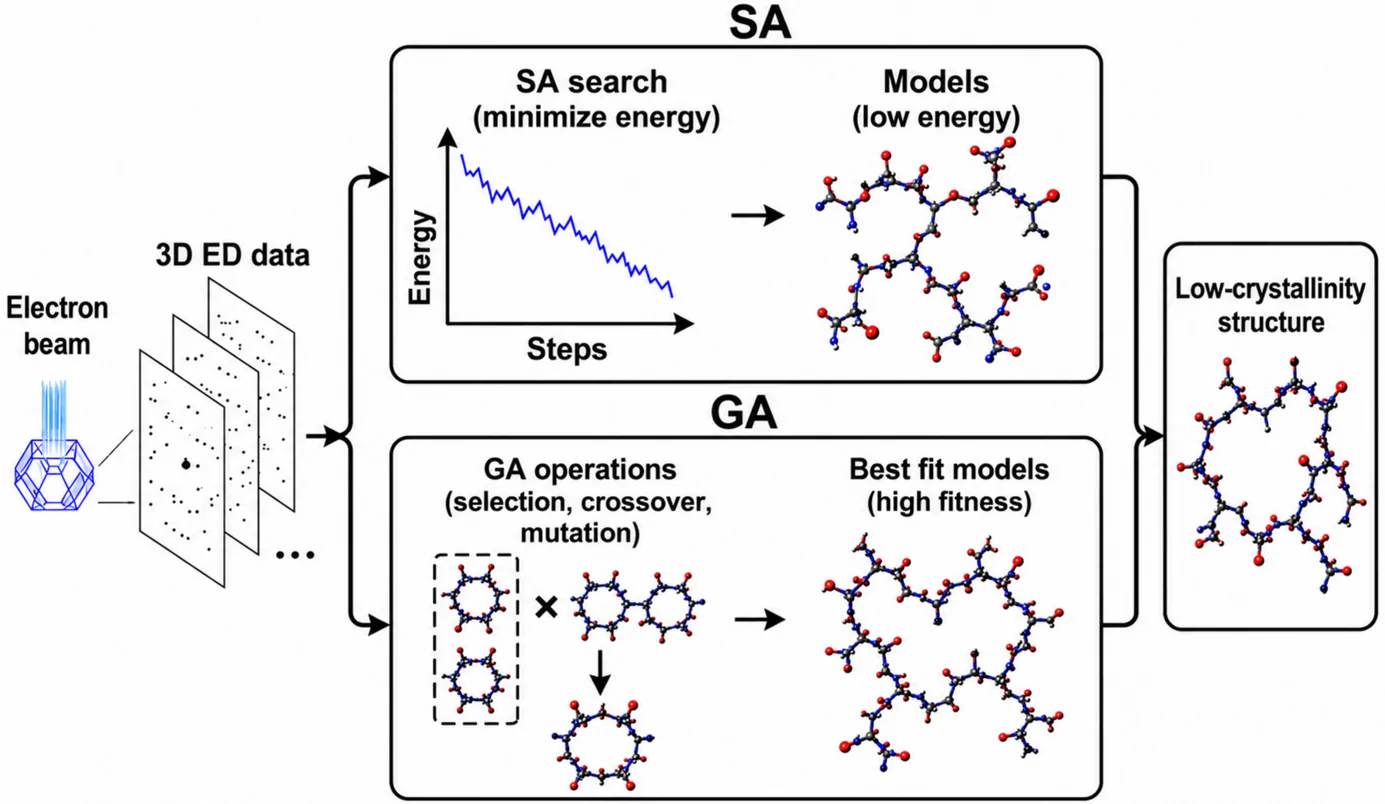
Schematic illustration of 3D ED approaches for structure determination of nanocrystals with low crystallinity.

In addition to SA, the direct-space genetic algorithm (GA) approach was found to successfully solve the structure of COF-300, showing an imine-linked 3D structure connecting tetra(4-amino­phenyl)­methane (TAPM) and terephthaldehyde building units, with a 3D ED data resolution of 3.78 Å (Sun *et al.*, 2020 ▸) [Fig. 5 ▸(*c*)]. Despite the low resolution, 3D ED data enabled the determination of the unit-cell parameters (*a* = *b* = 21.5 Å, *c* = 8.9 Å), reflection conditions and space group (*I*4_1_/*a*), which provided essential crystallographic information for defining the asymmetric unit and constructing chemically reasonable structural fragments. Recently, high-quality 3D ED data from COF-300 nanocrystals were obtained, which enabled *ab initio* structure determination at atomic resolution (Bourda *et al.*, 2024 ▸). Furthermore, computational intelligence strategies have also been integrated with electron crystallography for COF structure determination. By combining 3D ED with particle swarm optimization (PSO), which incorporates chemical constraints during structure generation and optimization, chemically feasible COF structures could be identified when diffraction data were limited by low crystallinity (Zhang, Hu *et al.*, 2025 ▸). These examples show that successful structure determination for poorly crystalline MOFs and COFs depends on 3D ED data resolution as well as appropriate structure-solution methods (Fig. 6 ▸).

### 2.2. Facilitating discovery of new materials

**Figure 7:**
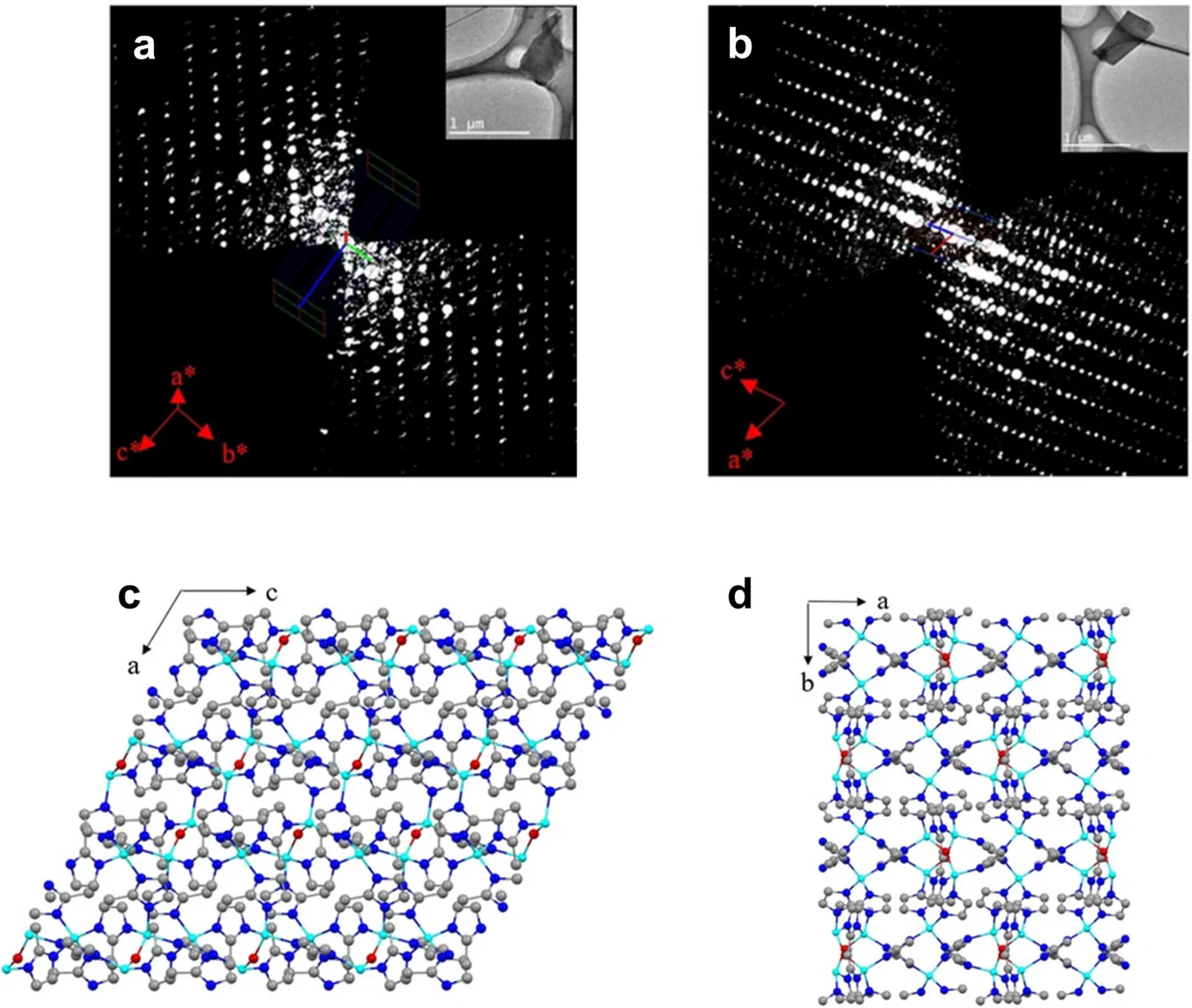
3D reconstructed reciprocal lattice of (*a*) ZIF-CO_3_-1 and (*b*) the newly discovered ZIF-EC1. The structural models of (*c*) ZIF-EC1 viewed along the *b* axis, and (*d*) ZIF-EC1 viewed along the *c* axis. Reprinted from Ge *et al.* (2021 ▸).

Owing to advances in reticular chemistry (Eddaoudi *et al.*, 2002 ▸), rational design has accelerated the development of novel MOFs and COFs that show unique properties related to their new structures (Huang *et al.*, 2016 ▸; Yuan, Qin, Lollar & Zhou, 2018 ▸; Jiang *et al.*, 2021 ▸; Huang, Willhammar & Zou, 2021 ▸; Liu *et al.*, 2024 ▸). However, in the search for novel MOFs and COFs, the synthesis can often result in a mixture of multiple phases. Without an unambiguous structural analysis, this often hinders the discovery of new materials. In addition, the minor phases in a mixture may easily be overlooked by conventional characterization methods. As the 3D ED method can complete the collection of a dataset within several minutes, it allows identification of hidden phases, and determination of each structure in a high-throughput manner (Ge *et al.*, 2021 ▸). A previously unknown MOF, ZIF-EC1 (INEVUV), was identified within a multiphase mixture by 3D ED. Although the amount of ZIF-EC1 in the phase mixture was less than 5%, its new structure was identified by 3D ED after analyzing more than 50 nanocrystals (Fig. 7 ▸). ZIF-EC1 possesses a dense 3D framework constructed from both Zn(II) cations and binuclear Zn clusters linked by 2-methylimidazolate (mIm) ligands. The high concentration of Zn and N species promoted the generation of abundant active sites during carbonization. In addition, the oxygen-containing structural moieties facilitated mesopore formation during pyrolysis, resulting in a porous architecture with high accessibility. Compared with other carbon materials derived from ZIFs such as NC-ZIF-1, NC-ZIF-8 and NC-ZIF-95, that derived from ZIF-EC1 showed the best catalytic performance for the oxygen reduction reaction. The high-throughput advantage of 3D ED is used to facilitate analysis of complex polycrystalline materials other than MOFs, such as pharmaceutical compounds (Lightowler *et al.*, 2024 ▸).

**Figure 8:**
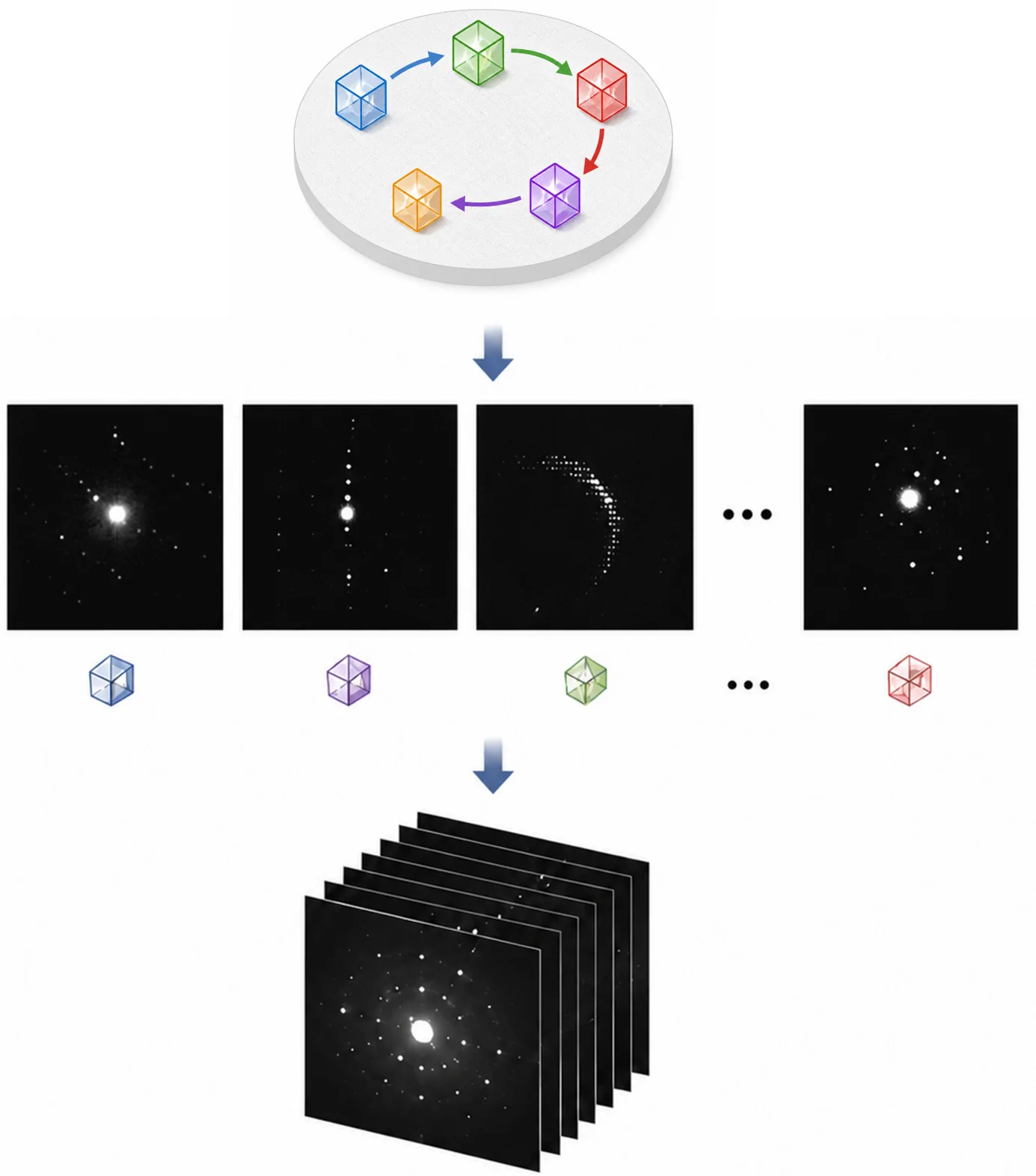
Schematic illustration of SerialED methods.

In addition to the advances mentioned above, serial electron diffraction (SerialED) has been developed as a high-throughput method for phase identification (Smeets *et al.*, 2018 ▸; Bücker *et al.*, 2020 ▸; Hogan-Lamarre *et al.*, 2024 ▸). SerialED enables high-throughput acquisition of a single electron diffraction pattern from each randomly oriented nanocrystal, without rotation of individual crystals. The resulting diffraction dataset is subjected to indexing and statistical analysis, providing crystallographic information for phase analysis (Fig. 8 ▸). Similarly, serial rotation electron diffraction (SerialRED) was developed by rotating each crystal during serial data collection, allowing acquisition of more complete reciprocal-space information. In combination with hierarchical cluster analysis, it was demonstrated in the structural analysis of PCN-416 (WONVIH). The structural models obtained showed that SerialRED could be used to obtain high-quality data comparable with manually collected 3D ED data, validating its reliability for automated structure determination (Wang *et al.*, 2019 ▸). In addition, SerialRED has also been used to analyze multiphase polycrystalline zeolite products (Luo *et al.*, 2023 ▸).

### 2.3. Study of host–guest interactions

After obtaining detailed structures, 3D ED has increasingly been applied to gain chemical insights into MOFs and COFs. Key questions in the study of porous materials are how guest molecules interact with the frameworks and how the frameworks respond during guest inclusion and removal. TEM imaging in real space has been used to analyze host–guest interactions in MOFs (Gong *et al.*, 2020 ▸). Compared with projected images, 3D ED provides information in 3D, making it possible to better locate guest species and understand host–guest interactions. For example, 3D ED was used to determine the precise locations of DABCO and water molecules within the pores of Co-CAU-36 (HIQDUJ), a porous cobalt phospho­nate MOF built from 1D inorganic chains of alternating CoO_4_ and PO_3_C tetrahedra. This revealed the hydrogen-bond networks between the DABCO molecules and the framework, and between the water molecules and the framework (Wang *et al.*, 2018 ▸). In a further example involving more complex organic molecules, 2-methyl-1,5-pentanedi­amine (MPMD) and tris­(2-amino­ethyl)-amine (TAEA) were identified in the pores of SU-8 (ZEJFUT) and SU-68 (ZEJGAA), respectively (Ge *et al.*, 2022 ▸). The atoms of the organic molecules were determined *ab initio* from 3D ED data along with the atoms from the frameworks. By directly locating the organic molecules, it was found that the same MPMD molecule resided in the pores of SU-8 with two different configurations. Moreover, it revealed the hydrogen-bonding interactions between organic molecules and the frameworks, as well as the van der Waals interactions between TAEA and SU-68. Through the distances between the guest molecules and frameworks, further insights regarding the strength of host–guest interactions at different sites can be obtained.

**Figure 9:**
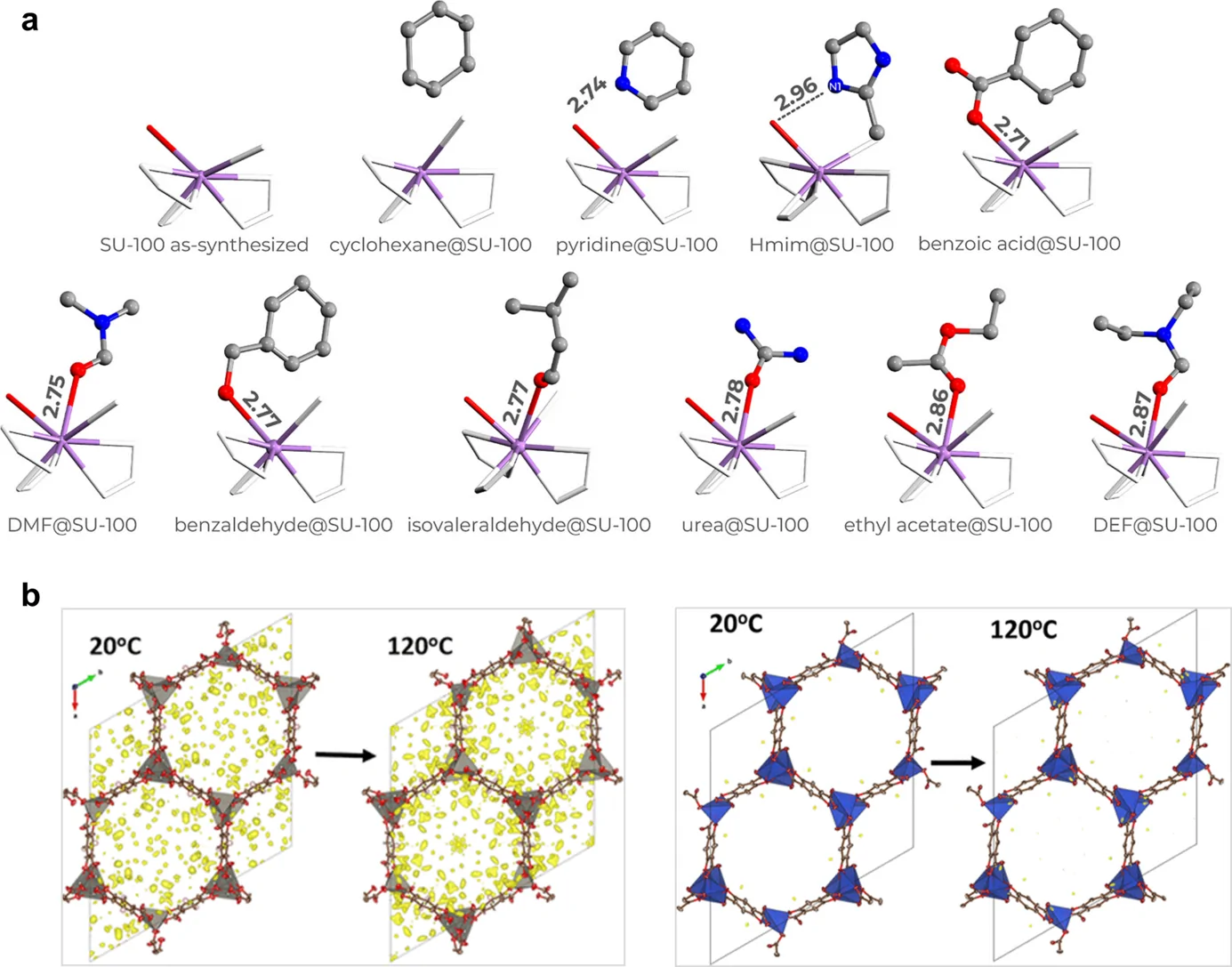
(*a*) Host–guest interactions between solvent molecules and the SU-100 framework. (*b*) Activation of Zn-MOF-74 (left) and Cu-MOF-74 (right) monitored by *in situ* heating 3D ED. Part (*a*) reprinted from Butonova *et al.* (2026 ▸) and part (*b*) reprinted from Quintelier *et al.* (2024 ▸).

While the study mentioned above was conducted at room temperature, locating guest molecules could require cryogenic temperatures, depending on the guest species and host frameworks. Under cryogenic temperatures, 3D ED was used for the direct location of pyridine, benzoic acid, 2-methyl­imidazole, benzaldehyde and cyclo­hexane within the pores of the bis­muth-based MOF SU-100 (OXIQAR), which is built from edge-sharing Bi–O inorganic building units and bi­phenyl-3,4′,5-tri­carboxyl­ate (BPT) linkers (Butonova *et al.*, 2026 ▸). Structural analysis revealed multiple interactions between the guest species and the host framework, including Bi—N coordination, hydrogen bonding, offset π–π stacking with aromatic linkers and van der Waals interactions. These interactions mediate the stabilization and periodic ordering of guest molecules within the porous framework [Fig. 9 ▸(*a*)].

3D ED has further been combined with *in situ* heating TEM to investigate DMF and water removal from Zn-MOF-74 and Cu-MOF-74, and the formation of open metal sites (Quintelier *et al.*, 2024 ▸). Zn-MOF-74 and Cu-MOF-74 were constructed from Zn(II) and Cu(II) metal centers with 2,5-di­hydroxy­terephthalic acid (H4dhtp) organic linkers. The activation process was monitored below 1.18 × 10^−5^ Pa with a stepwise heating protocol up to 350 °C. In Zn-MOF-74, it revealed that the coordinated DMF molecules were progressively removed upon heating, leading to the formation of open metal sites. Notably, residual electrostatic potentials were still detected within the pores after activation [Fig. 9 ▸(*b*)], suggesting that the removal of guest species was incomplete under such conditions. In contrast, Cu-MOF-74 exhibited few residual potentials following solvent exchange and exposure to ultrahigh vacuum, indicating that open metal sites could be generated more readily. The different activation pathways and thermal stabilities of the two materials were attributed to their distinct pore environments as the longer and weaker Cu—O bonds, the higher Lewis acidity of Cu centers and second-order Jahn–Teller distortions facilitated solvent removal.

### 2.4. Revealing framework flexibilities

**Figure 10:**
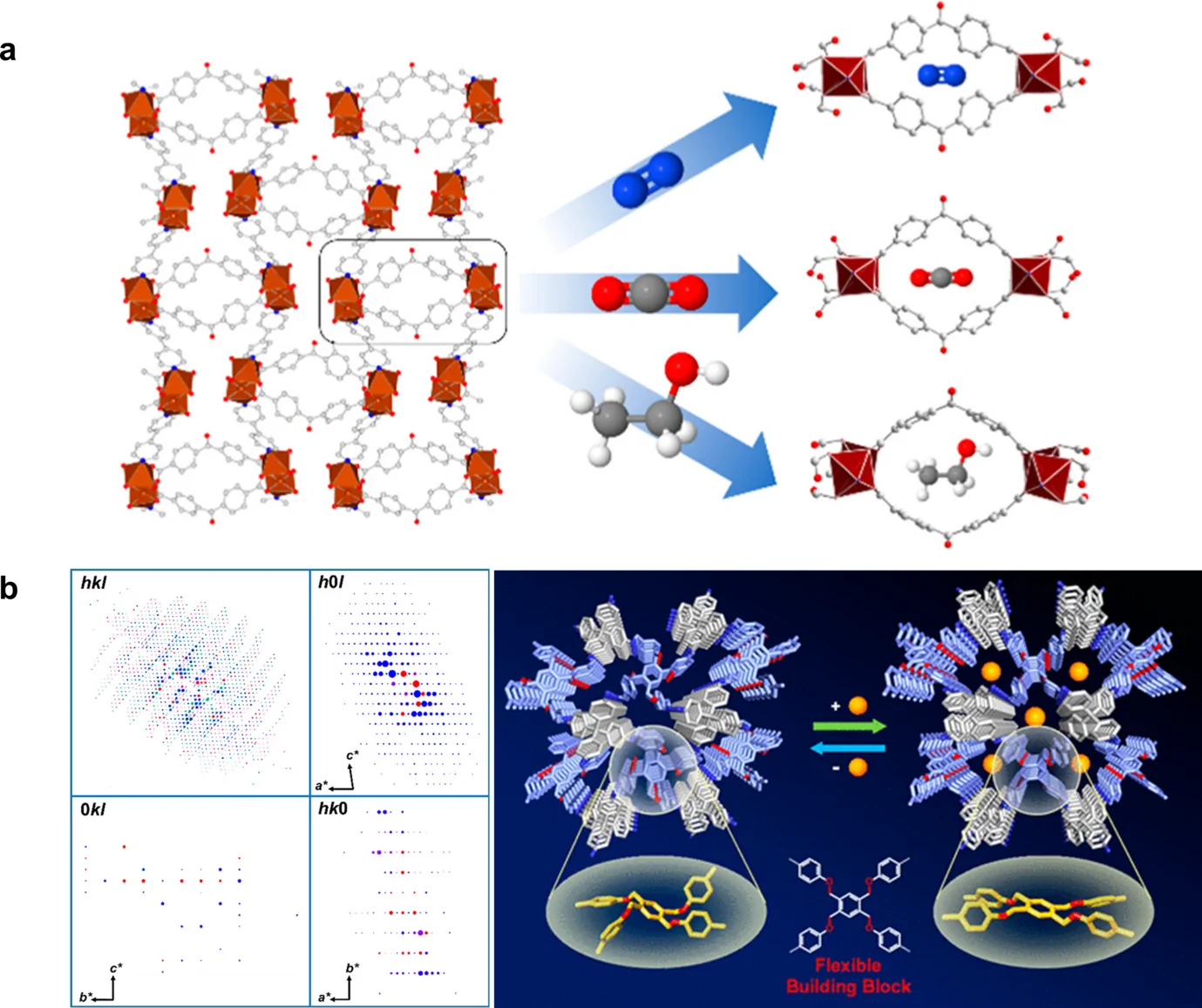
(*a*) Structural model of the Ru-MOF and guest-loaded N2@Ru-MOF, CO2@Ru-MOF and EtOH@Ru-MOF. (*b*) Structural models and corresponding 3D ED data for FCOF-5. Part (*a*) reprinted from Hindricks *et al.* (2024 ▸). Part (*b*) reprinted with permission from Liu *et al.* (2021 ▸). Copyright (2021) American Chemical Society.

In recent years, significant progress has been made in the field of flexible MOFs (Schneemann *et al.*, 2014 ▸; Bennett *et al.*, 2017 ▸; Wen *et al.*, 2025 ▸; Booth *et al.*, 2025 ▸). The flexibility of MOFs can be divided into two categories. Global flexibility refers to cooperative, framework-wide transformations, such as breathing, swelling, or network sliding, which are usually accompanied by changes in unit-cell parameters (Serre *et al.*, 2002 ▸; Smoljan *et al.*, 2025 ▸). In contrast, local flexibility arises from ligand motions or rearrangements of structural components, such as ligand conformational changes or local coordination distortions, without significant changes in the unit-cell parameters (Shivanna *et al.*, 2020 ▸). In addition, framework flexibility can be triggered in response to adsorption or desorption of guest molecules, which is important for gas separation and storage applications. The emergence of 3D ED has been used to reveal flexibilities of MOFs. For example, the structure and flexibility of a MOF, SU-100, were determined by 3D ED. 3D ED revealed that SU-100 underwent unit-cell parameter changes with different solvent molecules inside the pores. The solvent molecules affected the coordination geometry of the Bi^3+^ centers, particularly the Bi—O—Bi bond angles and Bi⋯Bi distances within the Bi_2_O_12_ dimeric inorganic building unit (IBU) of SU-100, leading to the breathing effect of the flexible MOF (Grape *et al.*, 2020 ▸). Furthermore, 3D ED was used to determine the structure of the flexible Ru-based MOF [Ru_2_(bzpdc)(bipy)] (bipy = 4,4′-bi­pyridine; bzpdc = 4,4′-benzo­phenone di­carboxyl­ate). It showed that the MOF was built from mixed-valent Ru_2_ paddlewheel IBUs (Hindricks *et al.*, 2024 ▸). The material undergoes reversible guest-induced narrow-pore to open-pore transformations upon adsorption of N_2_, CO_2_ and ethanol, mainly driven by distortion of the Ru_2_ paddlewheel units and conformational changes of the benzo­phenone-based bzpdc linker [Fig. 10 ▸(*a*)]. 3D ED showed that the ethanol-loaded phase retained the overall framework topology while undergoing substantial changes in lattice parameters and linker conformation.

3D ED has also been applied to study flexible COFs. An example is FCOF-5, which was synthesized by the [4 + 4] imine condensation of the flexible tetra-aldehyde linker 1,2,4,5-tetra­kis­[(4-formyl­phen­oxy)­methyl]­benzene (TFMB) and the rigid tetrahedral amine node TAPM. Using 3D ED, the structure was determined as a sixfold interpenetrated pts topology with flexible C—O single bonds in the framework backbone (Liu *et al.*, 2021 ▸). The framework was interconnected through C=N bonds, whereas the flexible C—O single bonds in the TFMB backbone provide the structural flexibility responsible for the breathing behavior. Upon THF vapor adsorption and desorption, the framework underwent reversible expansion and contraction driven by the conformational change of the flexible C—O linked building blocks, resulting in significant unit-cell volume variation [Fig. 10 ▸(*b*)]. Subsequent studies by 3D ED further revealed that the flexibility of the C—O bonds enables reversible framework expansion and contraction upon ethanol adsorption and desorption (Li *et al.*, 2022 ▸). Distinct gas-responsive structural flexibilities were also observed in atropisomeric COFs with different framework geometries. COF-320 (BOBHIL), constructed from tetra­kis­(4-amino­phenyl)­methane and 4,4′-bi­phenyl­dicarboxaldehyde, was determined by 3D ED to possess a ninefold interpenetrated dia framework with permanent porosity (Zhang *et al.*, 2013 ▸). 3D ED further identified an atropisomeric framework, COF-320-A (BOBHIL01), which possesses the same framework topology and degree of interpenetration but differs in the orientation of the imine bonds around the bi­phenyl linker (Kang *et al.*, 2023 ▸). In COF-320, the imine bonds point toward opposite sides, whereas in COF-320-A, they point toward the same side. Owing to the isomorphism, the structures exhibited different responses upon adsorption of N_2_, C_2_H_4_, CO_2_ and C_2_H_2_. COF-320 remained relatively rigid upon N_2_ adsorption, whereas COF-320-A underwent multiple adsorption-induced structural transformations, including pore expansion and crystal-to-crystal and crystal-to-amorphous transitions.

### 2.5. Probing dynamic structural behaviors

**Figure 11:**
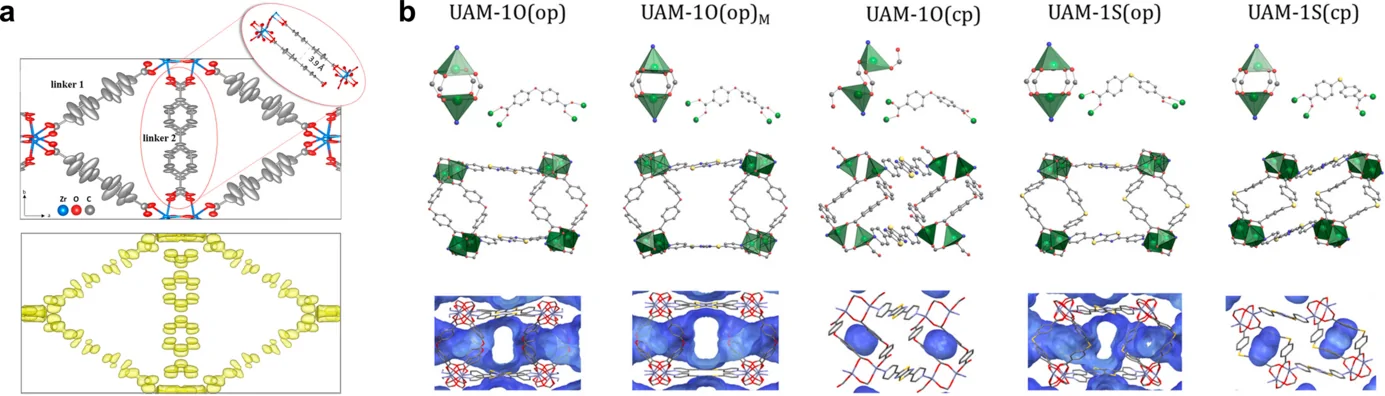
(*a*) Displacement ellipsoid model of MIL-140C obtained after refinement against 3D ED data and the electrostatic potential map. (*b*) Structural comparison of the open-pore (op) and contracted-pore (cp) phases of UAM-1O and UAM-1S. Part (*a*) reprinted from Samperisi *et al.* (2021 ▸) and part (*b*) reprinted from Sobczak *et al.* (2025 ▸).

The dynamic behaviors within MOF and COF structures are critical to the diffusion process of guest molecules, which could directly shed light at the atomic level for understanding framework evolution pathways and mechanisms during structural transformations. Recent developments in 3D ED have enabled direct probing of molecular motions in MOFs at the atomic level. For example, UiO-67 (WIZMAV14), a Zr-MOF constructed from Zr_6_O_4_(OH)_4_ oxo-clusters and 4,4′-bi­phenyldi­carboxyl­ate (bpdc) linkers, exhibited pronounced elongation of the displacement ellipsoids of the nonaxial carbon atoms, revealing dynamic motion of the bpdc linkers within the framework. In addition, MIL-140C (ZOGROF01), which is built from infinite Zr–oxo chains and bpdc linkers, showed different extents of motion of the bpdc molecules. The extents could be distinguished by analyzing the elongation of the displacement ellipsoids and the anisotropic displacement parameters derived from 3D ED data. It was found that the motions of isolated bpdc linkers were more pronounced than those of bpdc linkers reinforced by π–π stacking interactions [Fig. 11 ▸(*a*)]. This is because weak π–π interactions restrict linker motion and stabilize the framework, whereas isolated linkers possess greater spatial freedom and thus exhibit larger-amplitude motions (Samperisi *et al.*, 2021 ▸).

3D ED has also been extended from probing local linker motions to investigating framework-level dynamic responses in MOFs. A recent study using 3D ED compared two flexible isostructural MOFs, UAM-1S (CUVGUZ) and UAM-1O (CUVHAG), constructed from Zn paddlewheel units and a pillar ligand, 2,5-di(pyridin-4-yl)thia­zolo[5,4-d]thia­zole (TzTz), together with 4,4′-thio­dibenzoate (sba^2−^) and 4,4′-oxydibenzoate (oba^2−^), respectively. The two frameworks differed by only a single atom (S versus O) in the di­carboxyl­ate linker (Sobczak *et al.*, 2025 ▸). Despite this subtle structural difference, 3D ED showed that they exhibited markedly different dynamic transformation mechanisms [Fig. 11 ▸(*b*)]. UAM-1S underwent a continuous breathing process (second-order transition), whereas UAM-1O displayed a discrete gated mechanism involving Zn—O bond rearrangement and the formation of a metastable intermediate phase. By determining the atomic structures of the closed-pore states and identifying changes in metal-node connectivity, 3D ED provided direct structural evidence for the dynamic transformation pathways and enabled correlation of framework dynamics with local bonding rearrangements and phase-transition mechanisms.

Further studies show that the reliable analysis of molecular motions strongly depends on the quality of 3D ED data. Data completeness, resolution and signal-to-noise ratio can significantly affect the identification of linker motions, highlighting the necessity of obtaining high-quality 3D ED data for probing molecular motions in MOFs (Samperisi *et al.*, 2021 ▸; Wang & Huang, 2025 ▸).

3D ED is a valuable approach for studying dynamics not only in MOFs but also in COFs. The framework flexibility of COFs often gives rise to unique dynamic behavior (Chen *et al.*, 2019 ▸). Understanding the dynamics of COF-300 at the atomic level is particularly important due to its guest-responsive structural transformation, in which water adsorption induces significant framework contraction through cooperative hydrogen-bonding interactions within the pores (Ma *et al.*, 2018 ▸). The guest-dependent dynamics in COF-300 were directly revealed by 3D ED (Sun *et al.*, 2019 ▸). This showed that H_2_O adsorption led to unit-cell contraction, while the inclusion of ionic liquids and polymeric guests resulted in framework expansion. By locating both framework atoms and guest molecules, 3D ED showed that these dynamic transformations originate from geometric deformation of the tetrahedral nodes and conformational rearrangements of the imine-linked organic linkers. The rotational flexibility of the imine bonds acts as a molecular hinge, enabling framework contraction and expansion without significant bond stretching.

## 3. Conclusions and outlook

In summary, 3D ED has become a valuable and powerful method for structural analysis of MOFs and COFs. It not only enables structural determination of MOFs and COFs whose crystal sizes are too small for SCXRD, but also allows discovery of hidden phases, which can be overlooked in a phase mixture. Moreover, 3D ED can provide important structural information, such as stacking behavior of 2D MOFs and COFs, which is difficult to characterize by other methods. Recent advances in 3D ED have further expanded structural analysis of MOFs and COFs beyond their framework models to include host–guest interactions, framework flexibilities and structural dynamics. These developments show that 3D ED has evolved from a method primarily used for unknown structure determination into a valuable approach for gaining chemical insights and understanding structure–property relationships.

However, several challenges remain in the development of 3D ED. The applicability of 3D ED depends strongly on the crystallinity of the MOFs and COFs. Although 3D ED-based SA and GA have been successfully applied to solve COF structures with low resolution, the methods have not yet been applied to a wide range of poorly crystalline MOFs or COFs. Recent advances in artificial intelligence technologies could be combined with 3D ED to enhance the signal-to-noise ratio of data, improve data resolution of poorly crystalline MOFs and COFs, and boost the phasing process from low resolution data. In addition, further development in dynamical refinement for complex MOF and COF structures could result in more accurate structural determinations of these materials. Moreover, the emergence of *in situ* 3D ED methods could enable direct observation of MOF and COF structures under gas and liquid environments, providing insights into crystal growth, phase transitions, guest responsive behaviors and dynamic processes. We envision that the developments of 3D ED will provide further detailed structural insights and accelerate the research in the fields of MOFs and COFs.
